# Long-term treatment patterns and survival in metastatic breast cancer by intrinsic subtypes – an observational cohort study in Sweden

**DOI:** 10.1186/s12885-022-10098-1

**Published:** 2022-09-22

**Authors:** Henrik Lindman, Fredrik Wiklund, Klaus Kaae Andersen

**Affiliations:** 1 Uppsala University Hospital, SE-753 09 Uppsala, Sweden; 2 Statisticon AB, Uppsala, Sweden; 3 AstraZeneca Nordic, Copenhagen, Denmark

**Keywords:** Breast cancer, Treatment, Survival, Intrinsic subtypes, Clinical practice, Observational data, Cohort, Real world

## Abstract

**Background:**

Longitudinal, real-world data on the management of metastatic breast cancer is increasingly relevant to understand breast cancer care in routine clinical practice. Yet such data are scarce, particularly beyond second- and third-line treatment strategies. This study, therefore, examined both the long-term treatment patterns and overall survival (OS) in a regional Swedish cohort of female patients with metastatic breast cancer stratified by subtype in routine clinical practice during a recent eight-year period and correlation to current treatment guidelines.

**Methods:**

Consecutive female patients with metastatic breast cancer  clinically managed at Uppsala University Hospital, Uppsala, Sweden, during 2009–2016 and followed until the end of September, 2017 (*n* = 370) were included and, where possible, classified as having one of five, intrinsic subtypes: Luminal A; Luminal B; human epidermal growth factor receptor 2-positive (HER2+)/ estrogen receptor-positive (ER+); HER2+/estrogen receptor-negative (ER-); or triple negative breast cancer (TNBC). Treatment patterns and OS were estimated by subtype using time-to-event methods.

**Results:**

A total of 352/370 patients with metastatic breast cancer (mean age 67.6 years) could be subtyped: 118 (34%) were Luminal A, 119 (34%) Luminal B, 31 (8%) HER2+/ER-, 38 (11%) HER2+/Luminal, and 46 (13%) TNBC. The median number of metastatic treatment lines was 3. Most patients were on active treatment during follow-up (80% of the observation period), except for patients with TNBC who were on treatment for 60% of the observation time. Overall, 67% of patients died whilst on treatment. Among all patients (*n* = 370), median OS was 32.5 months (95% CI = 28.2–35.7). The 5-year survival rate was highest for HER2+/Luminal (46%) patients, followed by Luminal B (29%), Luminal A (28%), HER2+/ER- (21%), and TNBC (7%). Increasing age and number of metastatic sites also predicted worse survival.

**Conclusions:**

Metastatic breast cancer patients in Sweden, irrespective of subtype, generally receive active treatment until time of death. Survival varies considerably across subtypes and is also associated with patient characteristics. Regardless of differences in treatment patterns for Luminal A and B patients, long-term OS was the same.

**Supplementary Information:**

The online version contains supplementary material available at 10.1186/s12885-022-10098-1.

## Background

Breast cancer is the most common type of cancer in women, representing approximately 25% of all newly diagnosed cancers, and also the most common cause of death in women worldwide [1]. In Sweden, the incidence of breast cancer is increasing with over 7800 women diagnosed and 1400 women reported as dying with breast cancer as the underlying cause of death in 2017 [2]. When detected early, breast cancer generally has a favourable prognosis unlike metastatic disease; while treatable, metastatic breast cancer remains virtually incurable with an overall survival (OS) of approximately 3 years and a 5-year OS of only 25% [3]. Improved availability of increasingly effective systemic therapeutic modalities combined with earlier stage detection have had a positive effect on OS [2, 3]. Despite such advances in therapeutic options, management of patients with metastatic breast cancer still focuses primarily on prolonging survival and maintaining quality of life [4]. The health care system in Sweden is tax-based, providing equal therapy to all patients regardless of socio-economic status.

Treatment guidelines for metastatic breast cancer are generally complex and often appear to be difficult to implement in clinical practice. Current treatment strategies comprise systemic therapy (i.e., endocrine-, chemo-, and targeted-therapy) and local therapy. The choice of first-line therapy in the metastatic setting is based on tumour-related factors (e.g., the intrinsic tumour subtype) and a number of disease-related factors (e.g., disease-free interval, sites of recurrent disease, and prior treatment), as well as patient-related factors (e.g., performance status, comorbidities, and patient preference) [5].

Current guidelines state that patients with an estrogen receptor-positive (ER+)/human epidermal growth factor receptor 2-negative (HER2-) (Luminal) tumour subtype should be offered endocrine therapy, and if possible in combination with a cyclin-dependent kinase (CDK)4/6-inhibitor as first- (preferably) or second-line treatment, except for patients with visceral crises when chemotherapy is recommended [6]. In contrast, patients with a HER2-positive (HER2+) subtype should in first hand be treated using a combination of chemotherapy and trastuzumab and pertuzumab, where chemotherapy can be omitted or changed to endocrine therapy during the maintenance phase. Recommended second-line therapy for the HER2 subgroup of patients is trastuzumab-emtansine, with anti-HER2-targeted therapy continued after progression. In triple negative breast cancer (TNBC) patients, first-line chemotherapy is recommended. Recently, however, addition of immunotherapy with atezolizumab is an option in TNBC patients with Programmed Death Ligand 1-positive (PD-L1+) disease. There is a clear recommendation that biopsies from metastatic lesions should be analyzed as these may reveal changes in receptor expression and subsequently influence the choice of therapy [6].

Despite increasingly available treatment options, the continuously increasing incidence of metastatic breast cancer and the high number of women living with the disease little is still known about its management in routine clinical practice. This is particularly true for treatment beyond second- or third-line strategies, where detailed knowledge is scarce [6, 7]. Real-world data is important not only to improve actual disease management, but vital for the long-term prognosis of metastatic breast cancer.

In this observational study we examined the long-term treatment modality sequences and OS of metastatic breast cancer in clinical practice their relationship with current treatment guidelines using unique longitudinal observational data for all women managed for this disease regardless of intrinsic subtypes in a region of Sweden over an 8-year period.

## Methods

### Study design and data source

This observational cohort study was conducted using data from the RealQ® Breast Cancer clinical treatment database (Department of Oncology, Uppsala University Hospital, Uppsala, Sweden). The cohort included all female patients diagnosed with metastatic breast cancer at the hospital between 2009 and 2016 (including patients not receiving any therapy and de novo metastatic breast cancer patients). Patients were followed from the date of metastatic diagnosis (index) until death, migration or end of follow-up (30th September, 2017), whichever occurred first.

The database is well-established and validated regularly, with real-time collection of online data comprising detailed and high-quality information on diagnostics, treatment, and relapse from electronic medical records for all breast cancer patients diagnosed at the hospital. Data are added to the database at every specialist visit, and comprise data on tumour pathology, serum tumour markers, and individual cancer drugs (including supportive drugs, start- and stop-dates, clinical response, and reasons for discontinuation). Additionally, complete data from tumour scans, information on treatment side-effects (limited to chemotherapy), and patient symptoms, performance status, weight, and concomitant morbidity are included.

### Measures

Tumours were classified into five intrinsic subtypes based on a modified version of the St Gallen Guidelines [6] and recent Swedish data [8] according to surrogate marker panels using immunohistochemistry (IHC) and in situ hybridization (ISH). Luminal tumours were defined by ER+ (> 10%) and HER2- expression. Further sub-classification was based firstly on the Nottingham grade, where grades 1 and 3 were classified as Luminal A and B, respectively; secondly on high or low expression of Ki67 (where < 20% was defined as Luminal A and ≥ 20% as Luminal B); and, finally, in cases with grade 2 and no Ki67 result, a progesterone receptor-positive (PgR+) tumour (> 20%) was regarded as Luminal A and a PgR-negative (PgR-) tumour as Luminal B. Tumours that were HER2+ had to express IHC 3+ or 2+ with in-situ hybridization amplification and were sub-classified as HER2+/Luminal (ER+) and HER+/ER-. The TNBC tumours had to be ER-, PgR-, and HER2-. Patients in whom the intrinsic subtype could not be determined were defined as “Unclassified”.

The duration and composition of metastatic treatment regimens were assessed using dates and types of medications documented in the medical records, and categorized by chemotherapy, antibody therapy, endocrine therapy, and targeted therapy comprising mTOR-inhibitors, CDK4/6-inhibitors, and tyrosine kinase inhibitors.

Clinical data on patient characteristics were also obtained, specifically the date of incident breast cancer diagnosis, age, clinical staging at initial diagnosis, date of metastatic diagnosis, distant metastasis organ, and the Eastern Cooperative Oncology Group (ECOG) performance status (PS).

### Statistical analysis

Patient and disease characteristics at the first metastatic diagnosis (M1) stratified by different intrinsic subtypes were described as the mean, standard deviation (SD), median and interquartile range (IQR) for continuous variables, and counts and percentages for categorical variables. Overall survival was defined as the time from date of M1 until date of death or censoring, whichever occurred first.

Survival by cancer subtypes was estimated using Kaplan-Meier methods. Cox proportional hazards models were used to estimate adjusted hazard ratios for OS with 95% confidence intervals (CI). Treatment mapping to indicate each treatment period and treatment time was illustrated using a swimmer’s plot and color coding treatment categories. The proportion of time on active treatment during observation stratified by intrinsic subtype was visualized by estimated total person-time on treatment divided by total person-time at risk.

The statistical software R was used for all analyses [9].

## Results

### Patient characteristics

The study cohort included 370 female patients with metastatic breast cancer with a mean age of 7.2 years (SD13.6), and median time since primary diagnosis of 6 years. Of these patients, 57% had a performance status score of 0, with the dominant metastatic organs being visceral (66%) and bone (31%). A total of 352 of the 370 patients (95%) could be subtyped: 118 (32%) Luminal A, 119 (32%) Luminal B, 31 (8%) HER2+/ER-, 38 (10%) HER2+/Luminal, and 46 (12%) TNBC (Table 1). Diagnoses were based on biopsies of metastases in 143 of 352 patients (40.6%), and conversions of the basic subtype from the primary tumour occurred in 37 (26%) of these patients (Additional Table 1). Conversions from the primary Luminal to the HER2+ subtype and to TNBC occurred in 4 and 2% of patients, respectively. Four of the 26 primary HER2+ tumours had converted (Luminal metastasis), while 1 out of 10 (10%) of primary TNBC tumours changed their metastatic subtype (Luminal B).

**Table 1:** Patient characteristics at metastatic diagnosis, treatment lines received during follow up, and median survival by metastatic subtype

Patient characteristics at metastatic diagnosis (M1)							
	All	Luminal A	Luminal B	HER2+/ER-	HER2+/Luminal	Triple negative	Unclassified
	(*n* = 370)	(*n* = 118)	(*n* = 119)	(*n* = 31)	(*n* = 38)	(*n* = 46)	(*n* = 18)
**Age at M1 dx, years**							
mean (SD)	67.2 (13.6)	69.7 (12.2)	67.0 (12.9)	61.8 (15.4)	63.0 (15.4)	64.8 (13.2)	75.6 (13.6)
median (IQR)	67.6 (59.2–77.2)	69.1 (61.7–79.2)	69.0 (59.2–75.4)	62.0 (52.8–72.2)	65.3 (47.6–72.7)	64.5 (56.1–76.0)	80.7 (66.5–85.5)
< 50	50 (13.5%)	9 (7.6%)	18 (15.1%)	5 (16.1%)	12 (31.6%)	6 (13.0%)	0 (0.0%)
50–60	49 (13.2%)	13 (11.0%)	13 (10.9%)	8 (25.8%)	4 (10.5%)	8 (17.4%)	3 (16.7%)
60–70	105 (28.4%)	40 (33.9%)	31 (26.1%)	8 (25.8%)	7 (18.4%)	17 (37.0%)	2 (11.1%)
70–80	94 (25.4%)	31 (26.3%)	39 (32.8%)	7 (22.6%)	7 (18.4%)	7 (15.2%)	3 (16.7%)
80+	72 (19.5%)	25 (21.2%)	18 (15.1%)	3 (9.7%)	8 (21.1%)	8 (17.4%)	10 (55.6%)
**Time since primary dx, months**							
mean (SD)	72.0 (80.7)	87.2 (84.7)	66.2 (73.0)	28.7 (42.1)	46.5 (52.4)	52.3 (71.9)	189.6 (100.0)
median (IQR)	39.3 (7.0–109.4)	68.1 (6.3–128.5)	37.3 (11.5–94.9)	4.1 (0.7–39.2)	30.1 (3.2–63.2)	28.4 (13.1–42.0)	184.5 (145.0–232.3)
0–3	81 (21.9%)	29 (24.6%)	22 (18.5%)	15 (48.4%)	9 (23.7%)	5 (10.9%)	1 (5.6%)
3–12	21 (5.7%)	2 (1.7%)	8 (6.7%)	2 (6.5%)	4 (10.5%)	5 (10.9%)	0 (0.0%)
12–36	68 (18.4%)	9 (7.6%)	28 (23.5%)	3 (9.7%)	7 (18.4%)	21 (45.7%)	0 (0.0%)
36–60	40 (10.8%)	11 (9.3%)	12 (10.1%)	5 (16.1%)	7 (18.4%)	5 (10.9%)	0 (0.0%)
60+	160 (43.2%)	67 (56.8%)	49 (41.2%)	6 (19.4%)	11 (28.9%)	10 (21.7%)	17 (94.4%)
**Year at M1 dx, n (%)**							
2009–2011	157 (42.4%)	56 (47.5%)	51 (42.9%)	12 (38.7%)	9 (23.7%)	22 (47.8%)	7 (38.9%)
2012–2014	142 (38.4%)	38 (32.2%)	45 (37.8%)	16 (51.6%)	20 (52.6%)	17 (37.0%)	6 (33.3%)
2015–2016	71 (19.2%)	24 (20.3%)	23 (19.3%)	3 (9.7%)	9 (23.7%)	7 (15.2%)	5 (27.8%)
**Metastatic organs at M1 dx, n (%)**							
1	168 (45.4%)	52 (44.1%)	53 (44.5%)	15 (48.4%)	19 (50.0%)	19 (41.3%)	10 (55.6%)
2	110 (29.7%)	37 (31.4%)	39 (32.8%)	8 (25.8%)	9 (23.7%)	12 (26.1%)	5 (27.8%)
3+	92 (24.9%)	29 (24.6%)	27 (22.7%)	8 (25.8%)	10 (26.3%)	15 (32.6%)	3 (16.7%)
**Dominating site of disease at M1 dx, n (%)**^a^							
Visceral	244 (65.9%)	69 (58.5%)	72 (60.5%)	28 (90.3%)	30 (78.9%)	35 (76.1%)	10 (55.6%)
Bone	113 (30.5%)	47 (39.8%)	44 (37.0%)	2 (6.5%)	8 (21.1%)	4 (8.7%)	8 (44.4%)
Soft tissue	13 (3.5%)	2 (1.7%)	3 (2.5%)	1 (3.2%)	0 (0.0%)	7 (15.2%)	0 (0.0%)
**Performance status at M1 dx, n (%)**							
0	105 (57.4%)	33 (57.9%)	33 (52.4%)	12 (75.0%)	14 (66.7%)	11 (61.1%)	2 (25.0%)
1	56 (30.6%)	19 (33.3%)	21 (33.3%)	3 (18.8%)	5 (23.8%)	3 (16.7%)	5 (62.5%)
2	18 (9.8%)	4 (7.0%)	7 (11.1%)	0 (0.0%)	2 (9.5%)	4 (22.2%)	1 (12.5%)
3+	4 (2.2%)	1 (1.8%)	2 (3.2%)	1 (6.2%)	0 (0.0%)	0 (0.0%)	0 (0.0%)
Unknown	187 (50.5%)	61 (51.7%)	56 (47.1%)	15 (48.4%)	17 (44.7%)	28 (60.9%)	10 (55.6%)
**Adjuvant treatment before M1 Dx, n (%)**							
Endocrine therapy	201 (54.3%)	70 (59.3%)	79 (66.4%)	5 (16.1%)	22 (57.9%)	9 (19.6%)	16 (88.9%)
Radiotherapy	221 (59.7%)	67 (56.8%)	72 (60.5%)	14 (45.2%)	19 (50.0%)	34 (73.9%)	15 (83.3%)
Chemo therapy	154 (41.6%)	40 (33.9%)	50 (42.0%)	10 (32.3%)	15 (39.5%)	33 (71.7%)	6 (33.3%)
Antibody therapy	26 (7.0%)	1 (0.8%)	4 (3.4%)	7 (22.6%)	11 (28.9%)	3 (6.5%)	0 (0.0%)
**Treatment lines received following metastatic diagnosis**							
Number of treatment lines							
Mean (SD)	4.2 (3.2)	4.5 (3.4)	4.6 (3.4)	3.8 (2.6)	4.9 (3.0)	2.7 (2.5)	2.0 (1.5)
Median (IQR)	3.0 (2.0–6.0)	3.0 (2.0–7.0)	3.0 (2.0–7.0)	3.0 (2.0–5.0)	4.5 (3.0–6.8)	2.0 (1.0–4.0)	1.0 (1.0–3.5)
Range	0–18	1–18	0–15	0–10	0–13	0–9	0–5
**Proportion of time on active treatment following metastatic diagnosis by subtype**							
Treatment, median (IQR)							
Any	93 (75–98)	96 (89–99)	94 (83–99)	74 (43–91)	94 (83–98)	53 (19–76)	
Chemo	20 (0.0–55)	14 (0–48)	20 (0–51)	44 (16–79)	21 (2–62)	52 (19–73)	
Antibody	0 (0–0)	0 (0–0)	0 (0–0)	39 (15–70)	46 (16–76)	0 (0–0)	
Endocrine	55 (10–92)	72 (40–97)	66 (32–97)	0 (0–0)	56 (26–87)	0 (0–0)	
Targeted	0 (0–0)	0 (0–0)	0 (0–0)	0 (0–8)	0 (0–1)	0 (0–0)	
**Survival (months)**							
Death during follow-up, n (%)	258 (69.7%)	83 (70.0%)	79 (66.4%)	23 (74.1%)	19 (50.0%)	42 (91.3%)	12 (66.7%)
Median survival (95% CI)	33 (28–36)	43 (31–52)	36 (30–51)	31 (20–45)	42 (30-NA)	11 (8–18)	21 (12.7-NA)
2 yr OS (95% CI)	61 (56–66)	66 (58–76)	67 (59–76)	61 (46–81)	78 (66–93)	23 (13–39)	47 (29–79)
5 yr OS (95% CI)	26 (21–32)	28 (20–39)	29 (21–40)	21 (10–44)	46 (31–67)	7 (2–20)	25 (10–62)

^a^ Defined according to the following order: 1) visceral organs, 2) bone, and 3) soft tissue

Comparing patient characteristics across subtypes showed that patients with HER2+ or TNBC tumours were younger (median age 62 and 65 years, respectively) compared to those with Luminal A/B tumours. Patients with these tumour subtypes also had a shorter time between primary and metastatic diagnosis (median time 4 to 30 months for HER2+ or TNBC, respectively) than patients with Luminal A/B tumours (median time 37 to 68 months from primary diagnosis).

### Treatment patterns

**Fig. 1:**
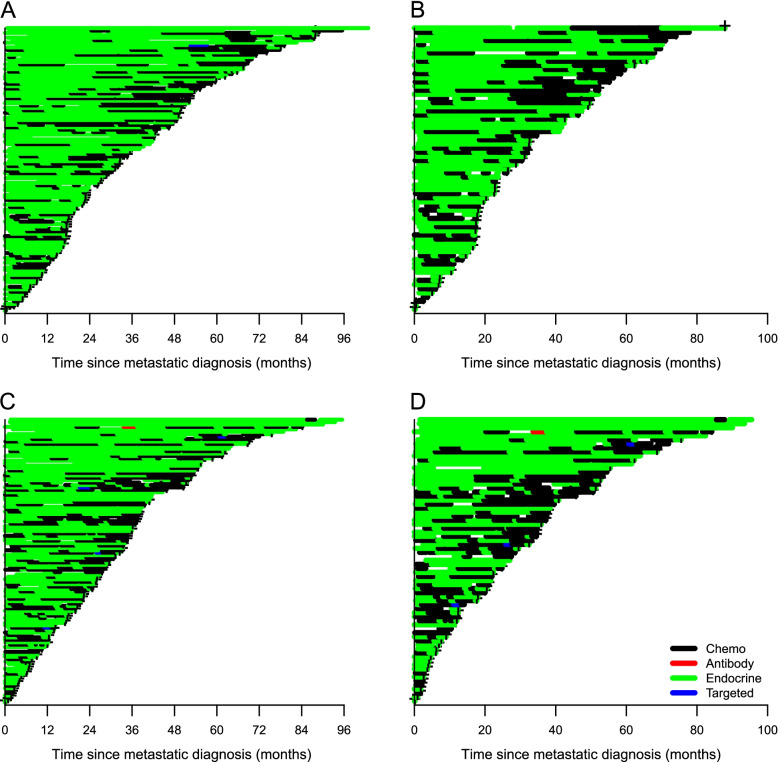
Treatment sequences for metastatic breast cancer patients: Luminal A, B, and Luminal B with visceral disease. The treatment sequences are described for metastatic breast cancer patients subtyped as Luminal A (**A**); Luminal A with visceral disease (**B**); Luminal B (**C**); and, Luminal B with visceral disease (**D**)

**Fig. 2:**
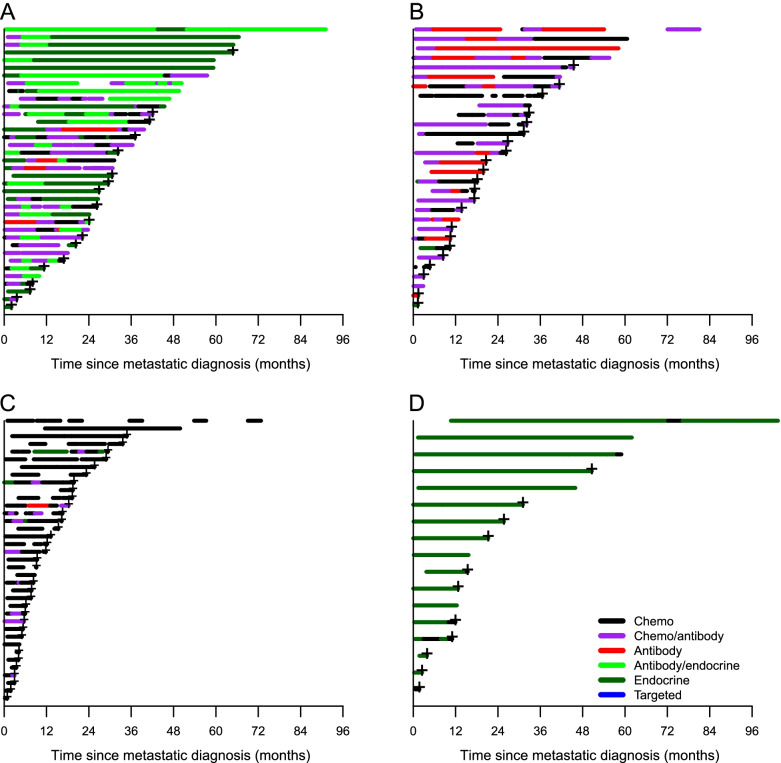
Treatment sequences for metastatic breast cancer patients: HER2/Luminal, HER2/ER–, TNBC; and, Unclassified. Treatment sequences for metastatic breast cancer patients: HER2/Luminal all patients (**A**); HER2/ ER– all patients (**B**); TNBC all patients (**C**); and, Unclassified (**D**), where HER = human epidermal growth factor receptor 2; ER– = estrogen receptor-negative; and, TNBC = triple negative breast cancer

Overall, 258 (70%) patients died during the observation period, of whom 13 (5%) were never initiated onto any therapy. The median number of metastatic treatment lines was 3 (Table 1). Most patients were on active treatment during follow-up (Additional Table 2) and 164 (67%) of the 245 patients receiving therapy died whilst on treatment. For patients not undergoing treatment at the time of death, the median time from the last treatment until death was 4.9 weeks (IQR = 1.4–13.6). Nearly all patients with Luminal A/B tumours received endocrine therapy followed by chemotherapy (Fig. 1). This treatment pattern was seen both for patients with and without visceral disease. Visual inspection of treatment patterns for HER2+ patients (Fig. 2) indicated that while HER2+/ER- patients mostly received either a combination of chemo/antibody or antibody therapy only (mainly trastuzumab regimens), several HER2+/ER+ patients had long treatment periods with endocrine therapy only. TNBC patients predominantly received chemotherapy; however, a few received antibody or chemotherapy/antibody combination therapy (bevacizumab), and a few received endocrine therapy despite being ER-. Patients without a metastatic tumour subtype classification received endocrine therapy.

### Survival

**Fig. 3:**
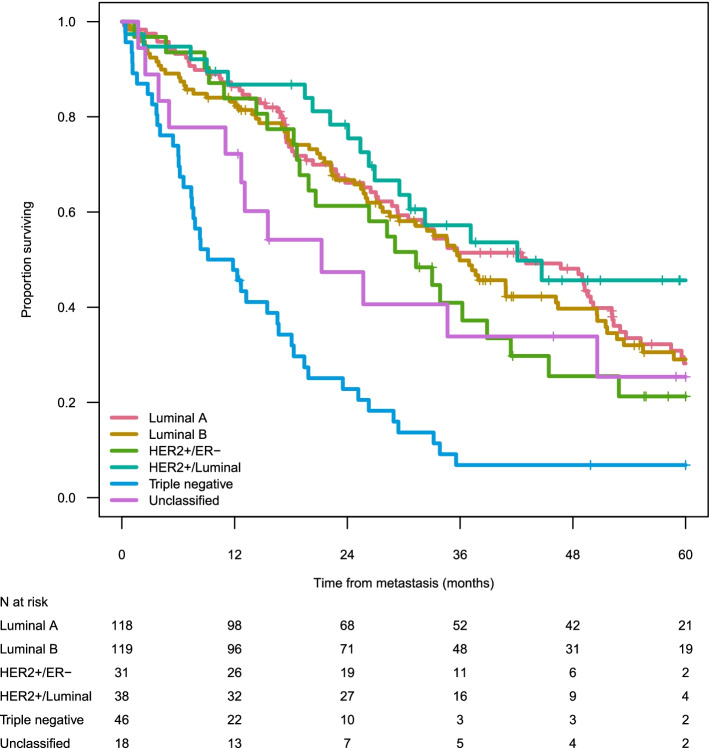
Unadjusted survival (Kaplan-Meier) by metastatic breast cancer subtype

When evaluating the entire patient cohort (*n* = 370), median OS was 32.5 months (95% CI = 28.2–35.7). The 5-year survival rate was highest for HER2+/Luminal patients (46%), followed by those with Luminal B (29%), Luminal A (28%), HER2+/ER- (21%), and TNBC (7%) tumour subtypes (Table 1, Fig. 3). Combining the HER2+ groups (HER2+/Luminal and HER2+/ER-) resulted in a median OS of 33.9 months (95% CI 29.1–52.9), and 2- and 5-years OS of 71% (0.60–0.82) and 33% (0.23–0.48), respectively.

Similarly, in the mutually adjusted Cox proportional hazards regression model, TNBC patients had an almost three-fold greater risk of all-cause mortality compared to those with other subtypes, HER2+/Luminal showing the lowest risk (Table 2). Age (> 80 years) and a metastatic pattern of 3+ metastatic sites were also independent risk factors significantly associated with increased mortality unlike time since primary diagnosis, dominant metastatic organ, and calendar time. A sensitivity analysis examining the effect of adjuvant treatment on survival did not show any association with mortality (*p* = 0.302).

**Table 2:** All-cause mortality expressed as crude and mutually adjusted hazard ratios (HR) with 95% confidence intervals (CI)

		Crude			Mutually adjusted		
	No. of patients/event	HR (95% CI)	***P*** value	P overall	HR (95% CI)	***P*** value	P overall
**Tumour subtype**							
Lum A	118/83	1.00			1.00		
Lum B	119/79	1.04 (0.76–1.41)	0.827		1.11 (0.80–1.53)	0.534	
HER2/ER neg	31/23	1.26 (0.79–2.01)	0.325		1.54 (0.93–2.54)	0.096	
HER2 Lum	38/19	0.76 (0.46–1.26)	0.292		0.73 (0.43–1.25)	0.254	
Triple negative	46/42	3.33 (2.27–4.86)	< 0.001	< 0.001	2.95 (1.85–4.70)	< 0.001	< 0.001
**Age at metastasis, years**							
< 50	50/32	1.00			1.00		
50–60	46/26	0.90 (0.54–1.51)	0.694		1.02 (0.60–1.75)	0.933	
60–70	103/77	1.02 (0.67–1.54)	0.935		0.99 (0.65–1.53)	0.972	
70–80	91/58	0.95 (0.62–1.47)	0.830		1.12 (0.71–1.76)	0.619	
80+	62/53	2.17 (1.40–3.37)	0.001	< 0.001	3.40 (2.12–5.47)	< 0.001	< 0.001
**No. of metastatic sites at M1**							
1	158/99	1.00			1.00		
2	105/76	1.19 (0.88–1.61)	0.247		1.39 (1.00–1.92)	0.051	
3+	89/71	1.71 (1.26–2.33)	0.001	0.003	1.98 (1.36–2.89)	< 0.001	0.002
**Dominating site of disease at M1 dx**							
Bone	105/63	1.00			1.00		
Soft tissue	13/12	2.06 (1.11–3.83)	0.022		1.24 (0.64–2.40)	0.533	
Visceral	234/171	1.48 (1.11–1.98)	0.007	< 0.001	1.11 (0.78–1.57)	0.560	0.755
**Time since primary diagnosis, months**							
0–3	80/50	1.00			1.00		
3–12	21/12	1.04 (0.55–1.95)	0.910		1.14 (0.59–2.20)	0.692	
12–36	68/53	2.02 (1.37–2.98)	< 0.001		1.77 (1.12–2.80)	0.015	
36–60	40/29	1.57 (0.99–2.48)	0.054		1.59 (0.99–2.54)	0.054	
60+	143/102	1.17 (0.83–1.64)	0.376	0.004	1.19 (0.83–1.70)	0.345	0.101
**Year at M1 diagnosis**							
2009–2011	150/131	1.00			1.00		
2012–2014	136/84	0.80 (0.60–1.06)	0.124		0.82 (0.61–1.10)	0.189	
2015–2016	66/31	1.33 (0.88–2.02)	0.178	0.055	1.42 (0.92–2.19)	0.112	0.056

### Luminal subtypes

Deceased Luminal B patients who had received treatment (*n* = 78) were younger (mean age 67.6 vs. 71.9 years), more commonly had 2+ metastatic sites (64.1% vs. 56.6%) and visceral as the primary metastatic location (69.2% vs. 59.0%) compared with deceased Luminal A patients who had received treatment (*n* = 83). Generally, Luminal B patients received chemotherapy more frequently than Luminal A patients, with chemotherapy more often administered during the later treatment lines (Fig. 1). The median proportion of time on active treatment with any chemotherapy (mono- or combinations of) was significantly greater for Luminal B patients than Luminal A patients (36% vs. 19%, respectively, *p* = 0.017). Moreover, for any endocrine therapy the median proportion of time treated was 50% for Luminal B patients vs. 68% for Luminal A patients (*p* = 0.29) (Fig. 2). There was no difference in mortality for Luminal B and A patients when adjusting for age, number of metastatic sites, visceral metastatic location, and treatment (*p* > 0.05) (Table 2).

### Treatment proportion

**Fig. 4:**
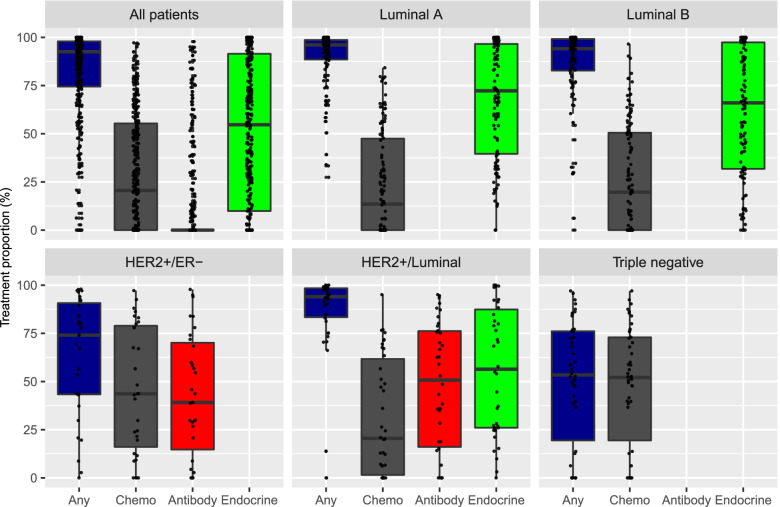
Proportion of time on active treatment during observation period by intrinsic subtype

Figure 4 illustrates the proportion of time on active treatment stratified by tumour subtypes. Patients with HER2+/ER- tumours had more time without treatment than the HER2+/Luminal subgroup (median 74.0 and 94.0% time on treatment, respectively). In contrast, patients with HER2+/Luminal tumours had less time on treatment with chemotherapy than the HER2+/ER- subgroup (median 20.5 and 43.6%, respectively). The HER2/Luminal patients had a numerically higher proportion of time on endocrine therapy than on (HER2-directed) antibody therapy, median 56.4 and 50.7% respectively.

## Discussion

This study using recent, real-world data shows that treatment patterns in metastatic breast cancer patients vary to a large extent. This supports the notion that guidelines for metastatic breast cancer are generally complex and often difficult to implement in clinical practice. We found that most Luminal A/B patients received endocrine therapy followed by chemotherapy in alignment with guideline recommendations [10], while HER2+ patients mostly received combinations of anti-HER2-targeted immunotherapies, with and without chemotherapy. Interestingly, treatment patterns for HER2+/ER+ patients indicate that several patients received endocrine maintenance therapy for long periods of time, and the proportion of time on endocrine treatment was higher than that on HER2-targeted immunotherapy. During the study period there was in fact some evidence to support such a treatment approach [11]. Moreover, the ABC Guidelines suggest that endocrine maintenance therapy following chemotherapy may be a reasonable option despite a lack of randomized data [10, 12]. The recommendation was based on clinical experience and associated lower toxicity [12] and further supported by the mechanistic rationale of cross-talk between estrogen- and HER2-receptors [13].

More recently, further evidence of endocrine maintenance therapy following chemotherapy/HER2-targeted antibody therapy for HER2+/ER+ patients has emerged [14, 15]. In the RegistHER and SystHER prospective registry studies, addition of endocrine therapy to chemotherapy and HER2-targeted therapy was associated with improved progression free survival and OS compared with chemotherapy and HER2-targeted therapy alone for HER+/ER+ patients [1, 15]. In our study, TNBC patients typically received chemotherapy; given the limited available treatment options and evidence to support the benefit of Programmed Cell Death (PD-1) and PD-L1-targeting antibodies during the observation period, this is not surprising. Nevertheless, a small number of patients received bevacizumab. Patients whom we were unable to classify based on tumour subtype received endocrine therapy. Surprisingly, our analyses reveal that a high number of HER2+ patients were managed for a long time without receiving HER2-targeted treatment. Currently we cannot explain this finding, but it does suggest that the younger age of these patients may have influenced these treatment patterns.

As regards survival, our study shows that the mortality rate for TNBC patients was about three times higher than for patients with all other subtypes, the latter all being associated with a comparable risk of death. This corresponds to the study of Li et al. [16], which also demonstrated that patients with TNBC had a worse overall survival than patients with non-TNBC independent of disease stage. Furthermore, we found that age (> 80 years) and 3+ metastatic sites were also independent risk factors for mortality, whereas time since primary diagnosis, dominating site of disease and calendar time were not statistically associated with increased mortality. Five-year survival was highest for HER2+/Luminal patients and median OS was 46 months. Similarly, in a French real-world study [17], HER2+ patients had a median OS of 45 months and also corresponded to the only metastatic subtype showing improvements in survival over calendar time. In our study, however, HER2+/ER- patients had a median OS of 31 months and, in contrast, no significant improvement in survival during the full study period despite a tendency to improved survival in the most recent years. This is in line with the French study [17] and also highlights the need for new treatment options. Notably, HER2+ patients were both younger and displayed a shorter time between primary and metastatic diagnosis than other subgroups.

### Luminal subtypes

Following the introduction of molecular subtyping of tumours in breast cancer [18], the Luminal B subtype has been associated with a worse prognosis than Luminal A breast cancer [19, 20]. Our data, however, suggest no difference in OS between Luminal A and Luminal B subgroups from the time of metastatic diagnosis, even when adjusting for prognostic factors. Since most previous reports on the prognostic impact of molecular subtypes contrary to our study analyse survival from time of diagnosis, the associated difference in survival outcomes we see may be associated primarily with a faster and higher prevalence of recurrence. Our study indicates that Luminal B patients were treated more often with chemotherapy and less often with endocrine therapy compared with Luminal A patients. This treatment approach is in line with suggested evidence that Luminal B breast cancer is more sensitive to chemotherapy, and less so to endocrine therapy than Luminal A breast cancer [21]. This is not, however, supported by guidelines for advanced breast cancer [6, 10, 12]. Conflicting results on the difference in survival of metastatic breast cancer between Luminal A and B patients have been reported previously. Two retrospective studies from Italy showed that patients with Luminal B breast cancer had a shorter time to progression when treated either with endocrine therapy and a CDK4/6 inhibitor [22] or with first-line endocrine therapy alone [23]. Another retrospective study from Japan showed that ER+ patients treated with first-line endocrine therapy showed no significant difference in survival from time of metastatic diagnosis whatever their Ki67 expression, whereas PgR expression was associated with improved survival [24].

Only a small proportion (< 10%) of patients were expected to be premenopausal, but unfortunately data on menopausal status was not available. Likely, menopausal status does not affect the differences observed between the Luminal A and B groups.

### Strengths and limitations

The major strength of our study is the detailed information on the diagnosis, treatment and follow-up of patients with metastatic breast cancer stratified by tumour subtype in a real-world setting with limited risk of selection bias. The completeness of the registry per se is high (approx. 95%) when validated with medical records. As this study was based on local data, however, the findings may not be directly generalizable to the entire advanced and/or metastatic breast cancer population in Sweden. Moreover, a number of limitations common to all observational studies apply here. Firstly, potential confounders have not been included, which may affect both internal and external validity of the results. This relates primarily to socio-economic factors that are known to be associated with breast cancer diagnosis [25], although access to healthcare is tax-financed in Sweden. Furthermore, lack of statistical power prevented us from describing specific treatment patterns within patient subgroups and investigating, for example, whether survival improved over time for HER2+ patients, as was shown by Gobbini et al. [17]. Our study reports on OS and treatment patterns. The focus for metastatic breast cancer remains prolonging survival and improving quality of life; however, the important quality of life evaluation was not possible using these data. Also, information on treatment side-effects, which may impact treatment patterns, could not be included.

## Conclusion

This observational study of metastatic breast cancer patients in clinical practice in Sweden shows that irrespective of tumour subtype, most patients (> 80%) were on active treatment until time of death. Median OS for metastatic breast cancer patients was 32.5 months, and 5-year survival rates varied considerably across subtypes. Regardless of Luminal A and B treatment differences, with larger share and earlier use of chemotherapy in the latter group, long-term OS was the same which could question the common recommendation of avoiding early use of chemotherapy in HR+/HER2- breast cancer. Also, encouraging survival data with CDK4/6 inhibitors or antibody-drug conjugates will be needed to be explored also in a real-world setting in the near future.

## Supplementary Information

**Additional file 1: Table 1.** Intrinsic subtype defined at primary diagnosis or re-biopsy.

**Additional file 2: Table 2.** Proportion of time on active treatment among patients by metastatic subtype.

## Data Availability

The data sources utilized in this study are all subject to local, ethical and privacy restrictions for data transfer abroad or into public domain limiting data availability on request. Any requests on data sharing may be directed to the corresponding author.

## Abbreviations

CDK: Cyclin-dependent kinase
CI: Confidence intervals
ECOG: Eastern Cooperative Oncology Group
ER-: Estrogen receptor-negative
ER+: Estrogen receptor-positive
HER2-: Human epidermal growth factor receptor 2-negative
HER2+: Human epidermal growth factor receptor 2-positive
HR: Hazard ratio
IHC: Immunohistochemistry
IQR: Interquartile range (IQR)
ISH: in-situ-hybridization
M1: First metastatic diagnosis
OS: Overall survival
PD-L1+: Programmed Death Ligand 1-positive
PgR-: Progesterone receptor-negative
PgR+: Progesterone receptor-positive
PS: Performance status
SD: Standard deviation
TNBC: Triple negative breast cancer

## References

[1] International Agency for Research on Cancer (IARC) – The Global Cancer Observatory. Globocan 2018. World Fact Sheet. http://gco.iarc.fr/today/data/factsheets/populations/900-world-fact-sheets.pdf. Accessed 21 March 2022.

[2] National Board of Health and Welfare. Statistics on Cancer incidence in 2017. Article no. 2018-12-51. https://www.socialstyrelsen.se/globalassets/sharepoint-dokument/artikelkatalog/statistik/2018-12-51.pdf. Accessed 21 March 2022.

[3] Cardoso F, Harbeck N, Fallowfield L, Kyriakides S, Senkus E (2012). ESMO guidelines working group. Locally recurrent or metastatic breast cancer: ESMO clinical practice guidelines for diagnosis, treatment and follow-up. Ann Oncol.

[4] Wörmann B (2017). Breast cancer: basics, screening, diagnostics and treatment. Med Monatsschr Pharm.

[5] Largillier R, Ferrero JM, Doyen J, Barriere J, Namer M, Mari V (2008). Prognostic factors in 1,038 women with metastatic breast cancer. Ann Oncol.

[6] Cardoso F, Senkus E, Costa A, Papadopoulos E, Aapro M, André F (2018). 4^th^ ESO-ESMO international consensus guidelines for advanced breast Cancer (ABC 4)†. Ann Oncol.

[7] Fietz T, Tesch H, Rauh J, Boller E, Kruggel L, Jänicke M (2017). Palliative systemic therapy and overall survival of 1,395 patients with advanced breast cancer - results from the prospective German TMK cohort study. Breast..

[8] Lundgren C, Bendahl PO, Borg Å, Ehinger A, Hegardt C, Larsson C (2019). Agreement between molecular subtyping and surrogate subtype classification: a contemporary population-based study of ER-positive/HER2-negative primary breast cancer. Breast Cancer Res Treat.

[9] R Core Team (2020). R:A language and environment for statistical computing. R Foundation for Statistical Computing, Vienna, Austria. URL https://www.R-project.org/. Accessed 21 March 2022.

[10] Cardoso F, Costa A, Norton L, Senkus E, Aapro M, André F (2014). ESO-ESMO 2^nd^ international consensus guidelines for advanced breast cancer (ABC2). Breast..

[11] Tripathy D, Kaufman PA, Brufsky AM, Mayer M, Yood MU, Yoo B (2013). First-line treatment patterns and clinical outcomes in patients with HER2-positive and hormone receptor-positive metastatic breast cancer from registHER. Oncologist..

[12] Cardoso F, Costa A, Senkus E, Aapro M, André F, Barrios CH (2017). 3^rd^ ESO-ESMO international consensus guidelines for advanced breast Cancer (ABC 3). Ann Oncol.

[13] Mehta A, Tripathy D (2014). Co-targeting estrogen receptor and HER2 pathways in breast cancer. Breast..

[14] Cobleigh M, Yardley DA, Brufsky AM, Rugo HS, Swain SM, Kaufman PA (2020). Baseline characteristics, treatment patterns, and outcomes in patients with HER2-positive metastatic breast cancer by hormone receptor status from SystHERs. Clin Cancer Res.

[15] Rimawi M, Ferrero JM, de la Haba-Rodriguez J, Poole C, De Placido S, Osborne CK (2018). First-line trastuzumab plus an aromatase inhibitor, with or without pertuzumab, in human epidermal growth factor receptor 2-positive and hormone receptor-positive metastatic or locally advanced breast cancer (PERTAIN): a randomized, open-label phase II trial. J Clin Oncol.

[16] Li X, Yang J, Peng L, Sahin AA, Huo L, Ward KC (2017). Triple-negative breast cancer has worse overall survival and cause-specific survival than non-triple-negative breast cancer. Breast Cancer Res Treat.

[17] Gobbini E, Ezzalfani M, Dieras V, Bachelot T, Brain E, Debled M (2018). Time trends of overall survival among metastatic breast cancer patients in the real-life ESME cohort. Eur J Cancer.

[18] Perou CM, Sørlie T, Eisen MB, van de Rijn M, Jeffrey SS, Rees CA (2000). Molecular portraits of human breast tumours. Nature..

[19] Sorlie T, Tibshirani R, Parker J, Hastie T, Marron JS, Nobel A (2003). Repeated observation of breast tumor subtypes in independent gene expression data sets. Proc Natl Acad Sci U S A.

[20] Cheang MC, Chia SK, Voduc D, Gao D, Leung S, Snider J (2009). Ki67 index, HER2 status, and prognosis of patients with luminal B breast cancer. J Natl Cancer Inst.

[21] Ades F, Zardavas D, Bozovic-Spasojevic I, Pugliano L, Fumagalli D, de Azambuja E (2014). Luminal B breast cancer: molecular characterization, clinical management, and future perspectives. J Clin Oncol.

[22] Palleschi M, Maltoni R, Ravaioli S, Vagheggini A, Mannozzi F, Fanini F (2020). Ki67 and PR in patients treated with CDK4/6 inhibitors: a real-world experience. Diagnostics (Basel).

[23] Rocca A, Farolfi A, Maltoni R, Carretta E, Melegari E, Ferrario C (2015). Efficacy of endocrine therapy in relation to progesterone receptor and Ki67 expression in advanced breast cancer. Breast Cancer Res Treat.

[24] Yamashita H, Toyama T, Nishio M, Ando Y, Hamaguchi M, Zhang Z (2006). p53 protein accumulation predicts resistance to endocrine therapy and decreased post-relapse survival in metastatic breast cancer. Breast Cancer Res.

[25] Dalton SO, Olsen MH, Johansen C, Olsen JH, Andersen KK (2019). Socioeconomic inequality in cancer survival - changes over time. A population-based study, Denmark, 1987-2013. Acta Oncol.

