# Supplementary material for: Long-term treatment patterns and survival in metastatic breast cancer by intrinsic subtypes – an observational cohort study in Sweden
Source: BMC Cancer. 2022 Sep 22;22:1006. doi: 10.1186/s12885-022-10098-1 (PMC9494782; doi:10.1186/s12885-022-10098-1)
Supplement: Supplementary file 1 — Additional file 1: Table 1. Intrinsic subtype defined at primary diagnosis or re-biopsy. [file 12885_2022_10098_MOESM1_ESM.docx]

**Additional Table 1:** Intrinsic subtype defined at primary diagnosis or re-biopsy

|  |  |  |  |  |
| --- | --- | --- | --- | --- |
|  | **All** | **Primary tumour subtype** | **Re-biopsy tumour subtype** |  |
| **Luminal A** | 118 (31.8%) | 68 (57.6%) | 50 (42.4%) |  |
| **Luminal B** | 119 (32.1%) | 74 (62.2%) | 45 (37.8%) |  |
| **HER2/ER-** | 28 (7.5%) | 11 (39.3%) | 17 (60.7%) |  |
| **HER2 Luminal** | 40 (10.8%) | 20 (50.0%) | 20 (50.0%) |  |
| **Triple negative** | 45 (12.1%) | 32 (71.1%) | 13 (28.9%) |  |
| **Unclassified** | 21 (5.7%) | 21 (100.0%) | 0 (0.0%) |  |
