# Supplementary material for: Long-term treatment patterns and survival in metastatic breast cancer by intrinsic subtypes – an observational cohort study in Sweden
Source: BMC Cancer. 2022 Sep 22;22:1006. doi: 10.1186/s12885-022-10098-1 (PMC9494782; doi:10.1186/s12885-022-10098-1)
Supplement: Supplementary file 2 — Additional file 2: Table 2. Proportion of time on active treatment among patients by metastatic subtype. [file 12885_2022_10098_MOESM2_ESM.docx]

| **Additional Table 2: Proportion of time on active treatment among patients by metastatic subtype** | | | | | | | | | |
| --- | --- | --- | --- | --- | --- | --- | --- | --- | --- |
|  | **Treatment exposure rate (%)** | | | | | | | | |
|  | **Any** | **Chemo** | **Antibody** | **Endocrine** | **Targeted** | **Che&Ant** | **Che&End** | **Ant&End** | **Che&Ant&End** |
| All patients (n=370) |  |  |  |  |  |  |  |  |  |
| Mean (SD) | 81.1 (25.6) | 29.4 (29.9) | 10.2 (23.5) | 51.3 (38.6) | 1.8 (6.1) | 6.4 (16.7) | 1.3 (8.0) | 2.6 (11.5) | 0.3 (4.0) |
| Median (IQR) | 92.5 (74.5-97.9) | 20.6 (0.0-55.4) | 0.0 (0.0-0.0) | 54.7 (9.9-91.5) | 0.0 (0.0-0.0) | 0.0 (0.0-0.0) | 0.0 (0.0-0.0) | 0.0 (0.0-0.0) | 0.0 (0.0-0.0) |
| Range | 0.0-100.0 | 0.0-97.2 | 0.0-97.8 | 0.0-100.0 | 0.0-50.2 | 0.0-95.1 | 0.0-81.3 | 0.0-90.6 | 0.0-71.8 |
| Lum A (n=118) |  |  |  |  |  |  |  |  |  |
| Mean (SD) | 89.5 (15.2) | 23.2 (26.7) | 1.1 (6.0) | 67.4 (30.2) | 1.2 (3.9) | 1.1 (6.0) | 1.1 (7.7) | 0.0 (0.0) | 0.0 (0.0) |
| Median (IQR) | 96.1 (88.5-98.7) | 13.5 (0.0-47.5) | 0.0 (0.0-0.0) | 72.3 (39.6-96.6) | 0.0 (0.0-0.0) | 0.0 (0.0-0.0) | 0.0 (0.0-0.0) | 0.0 (0.0-0.0) | 0.0 (0.0-0.0) |
| Range | 27.4-100.0 | 0.0-84.3 | 0.0-55.6 | 0.0-100.0 | 0.0-25.4 | 0.0-55.6 | 0.0-77.2 | 0.0-0.0 | 0.0-0.0 |
| Lum B (n=119) |  |  |  |  |  |  |  |  |  |
| Mean (SD) | 86.9 (19.1) | 26.6 (28.1) | 2.0 (8.2) | 61.6 (33.5) | 1.8 (6.4) | 1.9 (8.2) | 1.5 (9.4) | 0.0 (0.4) | 0.0 (0.0) |
| Median (IQR) | 94.1 (82.8-99.2) | 19.7 (0.0-50.5) | 0.0 (0.0-0.0) | 66.0 (31.8-97.4) | 0.0 (0.0-0.0) | 0.0 (0.0-0.0) | 0.0 (0.0-0.0) | 0.0 (0.0-0.0) | 0.0 (0.0-0.0) |
| Range | 0.0-100.0 | 0.0-96.6 | 0.0-65.1 | 0.0-100.0 | 0.0-50.2 | 0.0-65.1 | 0.0-81.3 | 0.0-4.4 | 0.0-0.0 |
| HER2/ER neg (n=31) |  |  |  |  |  |  |  |  |  |
| Mean (SD) | 63.5 (30.9) | 45.8 (32.9) | 42.8 (32.2) | 3.6 (15.1) | 6.6 (12.8) | 28.7 (27.5) | 0.0 (0.0) | 0.0 (0.0) | 0.0 (0.0) |
| Median (IQR) | 74.0 (43.4-90.7) | 43.6 (16.1-78.9) | 39.1 (14.7-70.1) | 0.0 (0.0-0.0) | 0.0 (0.0-7.8) | 26.8 (4.9-41.0) | 0.0 (0.0-0.0) | 0.0 (0.0-0.0) | 0.0 (0.0-0.0) |
| Range | 0.0-97.9 | 0.0-97.2 | 0.0-97.8 | 0.0-80.0 | 0.0-44.4 | 0.0-94.9 | 0.0-0.0 | 0.0-0.0 | 0.0-0.0 |
| HER2 Lum (n=38) |  |  |  |  |  |  |  |  |  |
| Mean (SD) | 86.7 (21.5) | 31.5 (30.3) | 47.4 (33.5) | 56.1 (33.1) | 2.5 (5.9) | 22.4 (25.2) | 3.9 (12.5) | 25.1 (27.3) | 3.2 (12.1) |
| Median (IQR) | 94.0 (83.4-98.4) | 20.5 (1.5-61.7) | 50.7 (16.1-76.2) | 56.4 (26.0-87.4) | 0.0 (0.0-0.2) | 13.7 (0.0-42.6) | 0.0 (0.0-0.0) | 16.8 (0.0-37.7) | 0.0 (0.0-0.0) |
| Range | 0.0-100.0 | 0.0-95.1 | 0.0-95.1 | 0.0-100.0 | 0.0-26.4 | 0.0-95.1 | 0.0-71.8 | 0.0-90.6 | 0.0-71.8 |
| Triple neg (n=46) |  |  |  |  |  |  |  |  |  |
| Mean (SD) | 49.7 (32.2) | 47.8 (31.1) | 6.6 (17.5) | 1.2 (5.6) | 0.1 (0.5) | 5.9 (16.6) | 0.0 (0.0) | 0.0 (0.0) | 0.0 (0.0) |
| Median (IQR) | 53.4 (19.4-76.1) | 52.1 (19.4-72.9) | 0.0 (0.0-0.0) | 0.0 (0.0-0.0) | 0.0 (0.0-0.0) | 0.0 (0.0-0.0) | 0.0 (0.0-0.0) | 0.0 (0.0-0.0) | 0.0 (0.0-0.0) |
| Range | 0.0-97.0 | 0.0-97.0 | 0.0-92.4 | 0.0-34.6 | 0.0-3.5 | 0.0-92.4 | 0.0-0.0 | 0.0-0.0 | 0.0-0.0 |
| **Treatment exposure proportion calculated on an individual level as the time exposed to treatment divided by total follow-up time** | | | | | | | | | |
